# Selective disruption of the E-cadherin–catenin system by an algal toxin

**DOI:** 10.1038/sj.bjc.6601640

**Published:** 2004-03-02

**Authors:** G Ronzitti, F Callegari, C Malaguti, G P Rossini

**Affiliations:** 1 Dipartimento di Scienze Biomediche, Università di Modena e Reggio Emilia, Via Campi 287, I-41100 Modena, Italy

**Keywords:** E-cadherin, *β*-catenin, *γ*-catenin, yessotoxin, N-cadherin, K-cadherin

## Abstract

Yessotoxins (YTXs) are algal toxins that can be accumulated in edible molluscs. YTX treatment of MCF-7 breast cancer cells causes the accumulation of a 100 kDa fragment of E-cadherin, which we have named ECRA_100_. A relative decrease in the concentrations of intact E-cadherin did not accompany the accumulation of ECRA_100_ in cytosoluble extracts of MCF-7 cells on the first day of YTX treatment, but a collapse of the E-cadherin system was detected after 2–5 days of treatment with the toxin. An analysis of the general structure of ECRA_100_ revealed that it consists of an E-cadherin fragment lacking the intracellular domain of the protein. ECRA_100_ was not released into culture media of YTX-treated cells. Accumulation of ECRA_100_ was observed in other epithelial cells, such as human intestine Caco-2 and MDCK cells after treatment with YTX. In turn, YTX could not induce accumulation of fragments of other members of the cadherin family, such as N-cadherin in the PC12 cell line and K-cadherin in sensitive cells (MCF-7, Caco-2, MDCK). The accumulation of a 100 kDa fragment of E-cadherin devoid of its intracellular domain induced by YTX was accompanied by reduced levels of *β*- and *γ*-catenins bound to E-cadherin, without a concomitant decrease in the total cytosoluble pools of *β*- and *γ*-catenins. Taken together, the results we obtained show that YTX causes the selective disruption of the E-cadherin–catenin system in epithelial cells, and raise some concern about the potential that an algal toxin found in seafood might disrupt the tumour suppressive functions of E-cadherin.

E-cadherin belongs to a large family of proteins responsible for the Ca^2+^-dependent cell–cell adhesion, which is involved in cell recognition, pattern formation and proper development of the embryo (Takeichi, 1990; Yap *et al*, 1997; Tepass *et al*, 2000). In adult vertebrates, E-cadherin is typically expressed in the cells of epithelia, where it plays a role of tumour suppressor (Vleminckx *et al*, 1991; Christofori and Semb, 1999), through the stabilisation of cell–cell adhesion by homophilic interactions between molecules projecting extracellularly from two contiguous cells (Vestweber and Kemler, 1985; Vleminckx *et al*, 1991; Christofori and Semb, 1999).

The proper functioning of E-cadherin depends on several portions of the protein molecule. The mature form of E-cadherin, in fact, consists of a transmembrane protein with an extended glycosilated amino-terminal exposed to the exterior of the cell, which contains the Ca^2+^-binding sites and mediates the homophilic adhesion. The carboxy-terminal, intracellular domain, in turn, is involved in supramolecular structures containing catenins and other cytosolic proteins, mediating proper attachment of E-cadherin to the actin cytoskeleton (Ozawa *et al*, 1989; Mareel *et al*, 1997; Christofori and Semb, 1999; Beavon, 2000; Nollet *et al*, 2000). Thus, alterations in the expression as well as in the structure of E-cadherin and catenins have been linked to tumour progression, tumour cell invasion and metastasis formation in breast, intestine and prostate cancers (Birchmeier and Behrens, 1994; Mareel *et al*, 1997; Christofori and Semb, 1999; Beavon, 2000).

Yessotoxins (YTXs) represent a group of polyether sulphated compounds (Yasumoto and Murata, 1993), which are produced by algae, such as *Protoceratium reticulatum* (Satake *et al*, 1997; Ciminiello *et al*, 2003). These natural compounds are included among algal toxins because i.p. injection of yessotoxin into mice leads to death at doses as low as 80 *μ*g kg^−1^ (Murata *et al*, 1987; Ciminiello *et al*, 1997; Ogino *et al*, 1997). As YTXs can be accumulated in edible molluscs, such as scallops and mussels (Murata *et al*, 1987; Ciminiello *et al*, 1997), by filter feeding, the current European legislation sets the limits of YTX contamination in material destined to human consumption (European Communities, 2002).

Oral ingestion of YTX at doses as high as 2 mg kg^−1^ has not led to mouse death and detection of severe organ damage (Aune *et al*, 2002; Tubaro *et al*, 2003), but ultrastructural alterations of cardiomyocytes have been observed by electron microscopy of tissue samples from mice that ingested high YTX doses (Tubaro *et al*, 2003). Long-term effects of YTX, in turn, have not been reported, yet.

In the course of our investigations onto the molecular bases of YTX action in cultured cells, we found that treatment of MCF-7 breast cancer cells with subnanomolar concentrations of YTX led to the accumulation of a 100 kDa fragment of E-caderin, which we have named ECRA_100_ (Pierotti *et al*, 2003).

Due to the possibility that disruption of E-cadherin functioning might favour tumour cell invasion and metastasis formation, and in the light of the very low effective concentrations of YTX in our experimental system, we have approached an analysis of the alterations induced by YTX in different cadherin molecules in cultured cells.

In this paper, we show that YTX causes the selective removal of the cytoplasmic domain of E-cadherin in epithelial cells, where it disrupts the E-cadherin–catenin system.

## MATERIALS AND METHODS

### Materials

Yessotoxin was obtained from the Institute of Environmental Science and Research Limited (Lower Hutt, New Zealand) and from Patrizia Ciminiello (Università di Napoli, Napoli, Italy).

Anti-E-cadherin antibodies were purchased from Santa Cruz Biotechnology (H-108), Transduction Laboratories (C20820) and Alexis Corporation (HECD-1). The anti-N-cadherin antibody was purchased from Assay Designs. The anti-K-cadherin antibody was purchased from Santa Cruz Biotechnology. The anti-*β*-catenin and anti-*γ*-catenin antibodies were obtained from Transduction Laboratories.

Peroxidase-linked anti-rabbit and anti-mouse Ig antibodies, the protein G-Sepharose^©^ and the enhanced chemioluminescence (ECL) detection reagents were from Amersham Biosciences, and the peroxidase-linked anti-goat Ig antibody was purchased from Santa Cruz Biotechnology.

The anti-actin antibody and prestained molecular mass markers were obtained from Sigma. The nitrocellulose membrane Protran BA 83 was obtained from Schleicher and Schuell. All other reagents were of analytical grade.

### Cell culture conditions and treatments

Cell cultures were grown in 5% carbon dioxide in air at 37°C, in 90-mm diameter Petri dishes. MCF-7 cells were obtained from the European Collection of Animal Cell Cultures (ECACC No. 86012803; CB No. CB 2705), and their culture medium was composed of Dulbecco's modified Eagle medium, containing 1% nonessential amino acids and 10% foetal calf serum.

Caco-2 cells were obtained from the American Type Culture Collection (ATCC No. HTB-37), their culture medium was composed of minimum essential medium with Earle's BSS, containing 2 mM glutamine, 2.2 g l^−1^ sodium bicarbonate, 1mM sodium pyruvate, 1% nonessential amino acids and 20% foetal calf serum.

PC-12 cells were obtained from the American Type Culture Collection (ATCC No. CRL-1721), and their culture medium was composed of RPMI 1640, containing 2 mM glutamine, 4.5 g l^−1^ glucose, 2 g l^−1^ sodium bicarbonate, 10 mM HEPES, 1 mM sodium pyruvate, 10% heat-inactivated horse serum and 5% foetal calf serum. These cells adhered poorly to plastic and tended to grow in small clusters. In our experimental conditions, attachment was improved by cell seeding in collagen-coated dishes (9 *μ*g collagen cm^−2^ solution). In some experiments, cell differentiation was stimulated by medium supplementation with 50 nM nerve growth factor (NGF) for 72 h at 37°C, according to Greene and Tischler (1976).

Madin Darby canine kidney (MDCK) cells were obtained from the American Type Culture Collection (ATCC No. CCL-34), and their culture medium was composed of minimum essential medium with Earle's salts, containing 2 mM glutamine, 1 mM sodium pyruvate, 1% nonessential amino acids and 10% foetal calf serum.

Stock solutions (1 *μ*M) of YTX were prepared by dissolving the compound in absolute ethanol, and were stored in glass vials protected from light at −20°C. If not stated otherwise, cell treatments were carried out using dishes near confluency, by addition of 1 nM YTX and incubations for 24 h at 37°C. Parallel dishes received the addition of absolute ethanol (control samples).

### Preparation of cell extracts

The experimental procedure was carried out at 2°C. If not stated otherwise, cells from culture dishes were washed once with PBS, harvested with PBS containing 1 mM EDTA and transferred to centrifuge tubes. MDCK cells were washed twice with PBS, and were then harvested by scraping. The cell suspensions were centrifuged for 8 min at 800 **g**, dispersed in PBS and centrifuged for 8 min at 800 **g**. The cell pellets were lysed with 0.5 ml PBS containing 1% (v v^−1^) Triton X-100 (TX buffer) and 0.1 mg ml^−1^ phenylmethylsulphonyl fluoride and with two 10 s bursts of vortexing. Cytosoluble extracts were then obtained by centrifugation for 30 min at 16 000 **g**. The supernatants of this centrifugation were then brought to 2% SDS and 5% *β*-mercaptoethanol, to be used for fractionation by sodium dodecyl sulphate-polyacrylamide gel electrophoresis (SDS–PAGE).

When the distribution of components between the Triton X-100-soluble and insoluble fractions was carried out, the precipitates obtained after the lysates had been centrifuged for 30 min at 16 000 **g** were extracted by dispersion with 20 mM Tris-HCl, pH 7.5 at 2°C, 2% SDS, 1 mM CaCl_2_, and were then subjected to centrifugation for 1 h at 10 5000 **g**. The supernatants of this centrifugation were brought to 5% *β*-mercaptoethanol and were used for SDS–PAGE.

The protein content of cellular extracts was measured with bicinchoninic acid (Smith *et al*, 1985).

When cell proliferation was evaluated, both adherent and floating cells were harvested and washed three times by resuspension in 4 ml of PBS, and low-speed centrifugation. The material remaining attached to the substratum was washed with PBS, was harvested with 20 mM Tris-HCl, pH 7.5 at 2°C, 1.5 mM EDTA (TE buffer) and was combined with its respective sediment. The resulting cellular suspension was then lysed by sonication with one 10 s burst, and the homogenate was used for DNA measurements by the procedure of Labarca and Paigen (1980).

### Immunoprecipitation of E-cadherin–catenin complexes

The samples used in this procedure consisted of cytosoluble extracts prepared as described, but lysing MCF-7 cells by a TX buffer containing a mixture of protease inhibitors including 2 mM 4-(2-aminoethyl)benzenesulphonyl fluoride, 1 mM EDTA, 130 *μ*M bestatin, 14 *μ*M E-64, 1 *μ*M leupeptin and 0.3 *μ*M aprotinin.

Cytosoluble extracts were pretreated with protein G-Sepharose^©^ for 1 h at 2°C. The treated extracts were recovered by low-speed centrifugation and were incubated with 1 *μ*g HECD-1 anti-E-cadherin antibody/700 *μ*g of cytosoluble protein for 1 h at 2°C. At the end of the incubation, 50 *μ*l of protein G-Sepharose^©^ was added to each sample and incubation was continued for 1 h at 2°C. The affinity matrix was then recovered by centrifugation for 4 min at 16 000 **g**, and was then washed twice by resuspension in 0.2 ml of PBS buffer, and centrifugation for 4 min at 16 000 **g** through a 0.5 ml cushion of 10% sucrose in PBS buffer. The material bound to the affinity matrix was then extracted with 100 *μ*l of TE buffer containing 4% (w v^−1^) SDS, 10 % *β*-MSH and 40 % (v v^−1^) glycerol, and was recovered by centrifugation for 4 min at 16 000 **g**. Samples were then subjected to SDS–PAGE.

### Fractionation of proteins by SDS–PAGE and immunoblotting

Samples containing the same amount of protein were fractionated by SDS–PAGE, according to Laemmli (1970), using a 10% separating gel and 3% stacking gel. After completion of electrophoresis, proteins were electrophoretically transferred onto a nitrocellulose membrane (Protran BA 83), and binding sites remaining on the membrane were blocked by incubation of blots for 1 h at room temperature with 20 mM Tris-HCl, pH 7.5 at 25°C, 0.15 M NaCl and 0.05% (v v^−1^) Tween 20 (immunoblotting buffer), containing 3% non-fat dry milk. When immunoblotting was used to detect cadherins, the immunoblotting buffers used for blocking unspecific sites on the membrane and for the incubation with primary antibodies did not include Tween 20, but contained 1 mM CaCl_2_. After blocking unspecific sites, the membranes were incubated for 1 h at room temperature with immunoblotting buffer, containing 1% non-fat dry milk and the primary antibody at a final concentration ranging between 0.1 and 2 *μ*g ml^−1^, depending on the antigen to be detected. After incubation, membranes were washed five times with immunoblotting buffer, and incubated for 1 h at room temperature with a peroxydase-linked secondary antibody at a 1 : 3000 or 1 : 2000 dilution in immunoblotting buffer containing 1% non-fat dry milk. After washing, the membranes were developed by the ECL detection system, and the results were visualised by autoradiography. The results, shown in figures, are representative of those obtained in the three or more independent experiments performed.

## RESULTS

**Figure 1:**
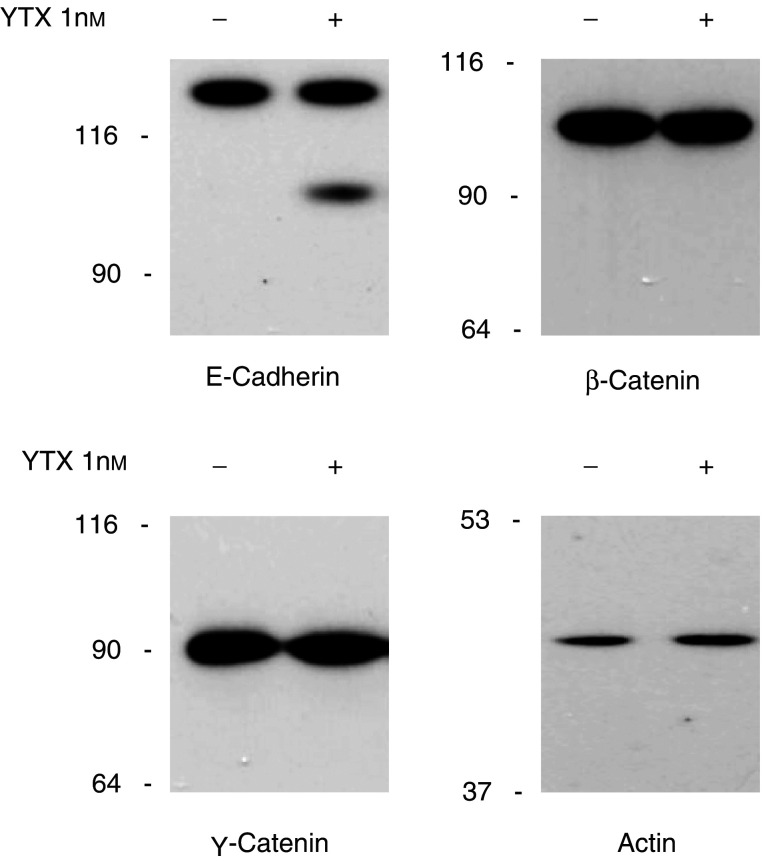
Effect of yessotoxin treatment of MCF-7 cells on the components of the E-cadherin–catenin system. Cells were incubated with (+) or without (−) 1 nM YTX for 24 h at 37°C. At the end of the incubation, cells were processed to prepare cytosoluble extracts, which were subjected to SDS–PAGE and immunoblotting, using antibodies recognising the indicated proteins. Extracts loaded onto each lane contained 10 *μ*g of protein, and detection of actin has been included as a loading control for our procedure. The electrophoretic mobilities of *β*-galactosidase (116 kDa), fructose-6-phosphate kinase (90 kDa), pyruvate kinase (64 kDa), fumarase (53 kDa) and lactate dehydrogenase (37 kDa) subunits, used as marker proteins running in a parallel lane, are indicated on the left.

The original observation that YTX treatment induces fragmentation of E-cadherin in MCF-7 cells (Pierotti *et al*, 2003) led us to ascertain preliminarily whether similar structural alterations could be detected in components associated with E-cadherin, such as *β*- and *γ*-catenins (Ozawa *et al*, 1989; Mareel *et al*, 1997). By immunoblot analysis of cytosoluble extracts, we could observe that neither the cellular levels nor the general structures of *β*- and *γ*-catenins were significantly changed after MCF-7 cells had been treated for 24 h with YTX, when ECRA_100_ was accumulated (Figure 1

).

**Figure 2:**
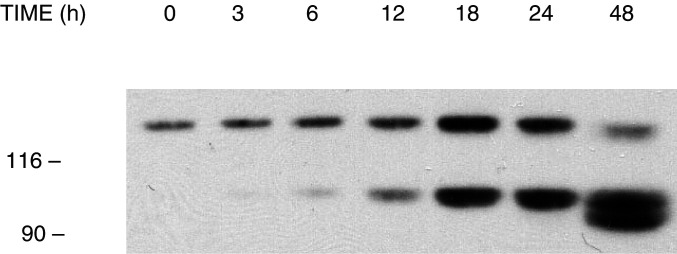
Time-course of the fragmentation of E-cadherin induced by yessotoxin in MCF-7 cells. Cells in logarithmic growth were incubated with 1 nM YTX for the times indicated at 37°C. At the end of the incubation, cells were processed to prepare cytosoluble extracts, which were subjected to SDS–PAGE and immunoblotting, using the HECD-1 anti-E-cadherin antibody. The electrophoretic mobilities of *β*-galactosidase (116 kDa) and fructose-6-phosphate kinase (90 kDa) subunits, used as marker proteins running in a parallel lane, are indicated on the left.

Other experiments were then carried out to define the temporal pattern of E-cadherin fragmentation in YTX-treated MCF-7 cells. To this end, cultures were treated with 1 nM YTX for different times before extracts were prepared and analysed by immunoblotting, using an anti-E-cadherin antibody (Figure 2

). The results we obtained confirmed our initial observation that very low levels of ECRA_100_ are detected in control cells (Pierotti *et al*, 2003). An increase in the concentrations of ECRA_100_ in cytosoluble extracts became detectable after a 6 h treatment with YTX, reaching maximal levels 18–24 h after toxin addition, whereas other fragments were detected thereafter (Figure 2).

**Figure 3:**
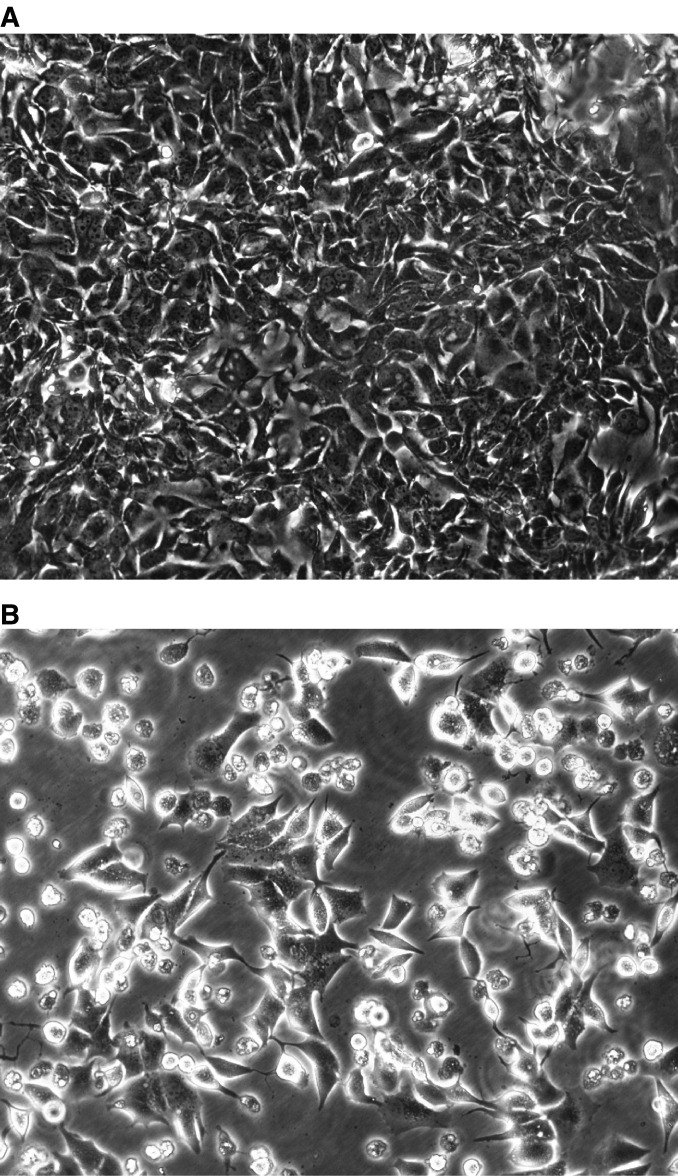
Effect of yessotoxin on morphology of MCF-7 cells in culture. Phase contrast microscopy (magnification × 200) of MCF-7 cells after treatment for 4 days with vehicle (**A**) or 1 nM YTX (**B**).

In line with previous data (Pierotti *et al*, 2003), a relative decrease in the concentrations of intact E-cadherin did not appear to accompany the accumulation of ECRA_100_ in cytosoluble extracts of MCF-7 cells in the first day of YTX treatment (Figure 2). However, a prolonged incubation with YTX eventually led to a collapse of the E-cadherin system in MCF-7 cells. In the experiments we have carried out so far, this collapse involved 70–90% losses in the cellular levels of intact E-cadherin that was detected between 2 and 5 days after YTX addition to cultured cells, and was accompanied by cell detachment from culture dishes (Figure 3

).

**Figure 4:**
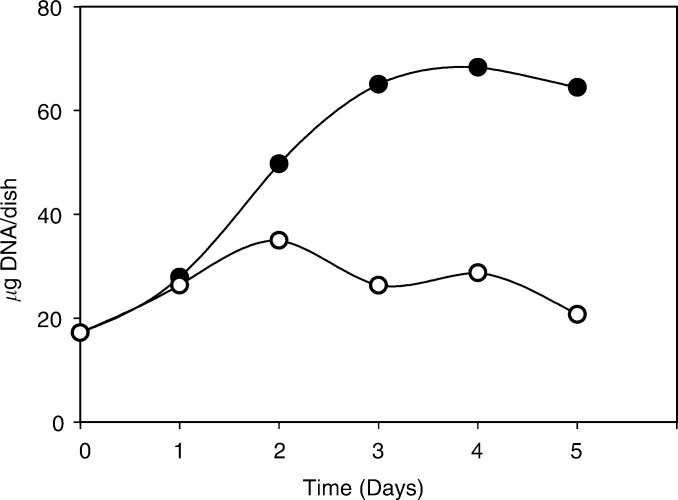
Effect of yessotoxin on the proliferation of MCF-7 cells. Cells were treated with either 1 nM yessotoxin (empty circles) or vehicle (filled circles), and cultures were maintained for the indicated times at 37°C, before being used for measurements of DNA content, as described under Materials and Methods.

This finding led us to analyse the effect of YTX on MCF-7 cell proliferation, and we found that cell growth apparently ceased after 24–48 h of treatment with YTX (Figure 4

). Extensive MCF-7 cell death, however, did not occur, as the cell content of YTX-treated cultures did not drop in the following days (Figure 4).

The immunoblot analysis of E-cadherin described above was performed using the HECD-1 antibody, whose epitope is located at the amino-terminal in the extracellular domain of the protein (Shimoyama *et al*, 1989), supporting the hypothesis that ECRA_100_ lacks the carboxy-terminal, intracellular, portion of the molecule.

**Figure 5:**
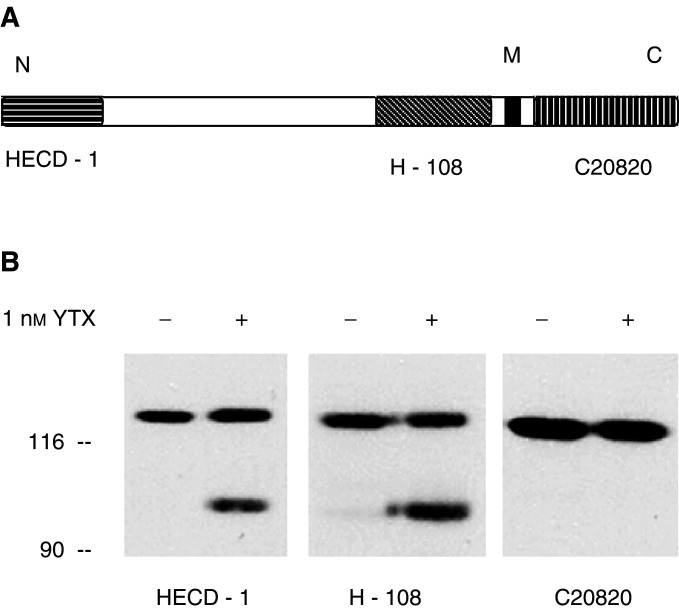
Characterisation of ECRA_100_ by immunoblotting. (**A**) Schematic representation of the E-cadherin molecule, including the transmembrane segment (in black) and the localisation of the epitopes bound by the HECD-1, H-108 and C20820 anti-E-cadherin antibodies. (**B**) MCF-7 cells were incubated with 1 nM YTX for 24 h at 37°C. At the end of the incubation, cells were processed to prepare cytosoluble extracts, which were subjected to SDS–PAGE and immunoblotting, using the HECD-1, H-108 and C20820 anti E-cadherin antibodies, as indicated. The electrophoretic mobilities of *β*-galactosidase (116 kDa) and fructose-6-phosphate kinase (90 kDa) subunits, used as marker proteins running in a parallel lane, are indicated on the left.

An analysis of the general structure of ECRA_100_ was then approached by antibodies whose epitopes are located in different domains of the E-cadherin molecule (Figure 5A

). H-108 is a polyclonal anti-E-cadherin antibody raised against a recombinant protein corresponding to amino acids 600–707, mapping within the extracellular domain of the protein of human origin (see the information sheet of the product (Cat. No. sc-7870) provided by Santa Cruz Biotechnology). When immunoblot analysis was carried out with the H-108 anti-E-cadherin antibody, the electrophoretic patterns we detected did not differ from those found with the HECD-1 antibody (Figure 5B) The monoclonal C20820 anti-E-cadherin antibody, in turn, whose epitope consists of the carboxy-terminal, intracellular domain of human E-cadherin (see the information sheet of the product (Cat. No. C20820) provided by transduction Laboratories), bound the intact protein, but could not interact with ECRA_100_ (Figure 5B).

According to these findings, therefore, ECRA_100_ consists of an E-cadherin fragment lacking some of the intracellular domain of the protein.

Based on these features, we checked whether ECRA_100_ might be released from plasma membrane when MCF-7 cells were treated with YTX. By immunoblot analysis of proteins obtained from culture media of YTX-treated cells, however, we could not detect ECRA_100_, even after the protein concentration of our samples was concentrated 110-fold by ultrafiltration (data not shown).

**Figure 6:**
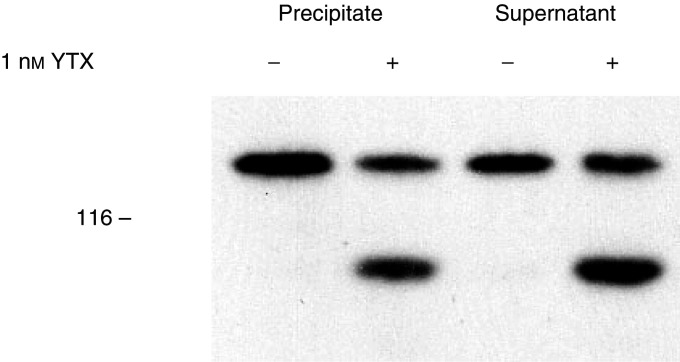
Effect of yessotoxin on the distribution of E-cadherin and ECRA_100_ between soluble and particulate material prepared from MCF-7 cells. Cells were incubated with 1 nM YTX for 24 h at 37°C, before being processed to prepare Triton X-100 soluble (supernatant) and insoluble (precipitate) components, as described under Materials and Methods. Samples were then subjected to SDS–PAGE and immunoblotting, using the HECD-1 anti-E-cadherin antibody. The electrophoretic mobility of *β*-galactosidase (116 kDa) subunits, used as marker proteins running in a parallel lane, is indicated on the left.

The possibility that ECRA_100_ might partition only in the Triton X-100 soluble fraction was also evaluated by preparing soluble and insoluble fractions from control and YTX-treated cells, as described in the Materials and Methods section. By immunoblot analysis of the two fractions, we ascertained that part of cellular ECRA_100_ is resistant to nonionic detergents, and is detectable in the Triton X-100 insoluble fraction prepared from YTX-treated cells (Figure 6

).

The intracellular carboxy-terminal domain of E-cadherin contains the binding sites for *β*- and *γ*-catenins (Ozawa *et al*, 1989; Stappert and Kemler, 1994; Jou *et al*, 1995). Since YTX induces structural alterations of this portion of E-cadherin, we ascertained whether it could also affect the association of *β*- and *γ*-catenins with E-cadherin. To this end, we immunoprecipitated E-cadherin using the HECD-1 anti-E-cadherin antibody, and measured the levels of *β*- and *γ*-catenins associated with E-cadherin under our experimental conditions, by SDS–PAGE and immunoblot analysis of components found in the immunoprecipitates.

**Figure 7:**
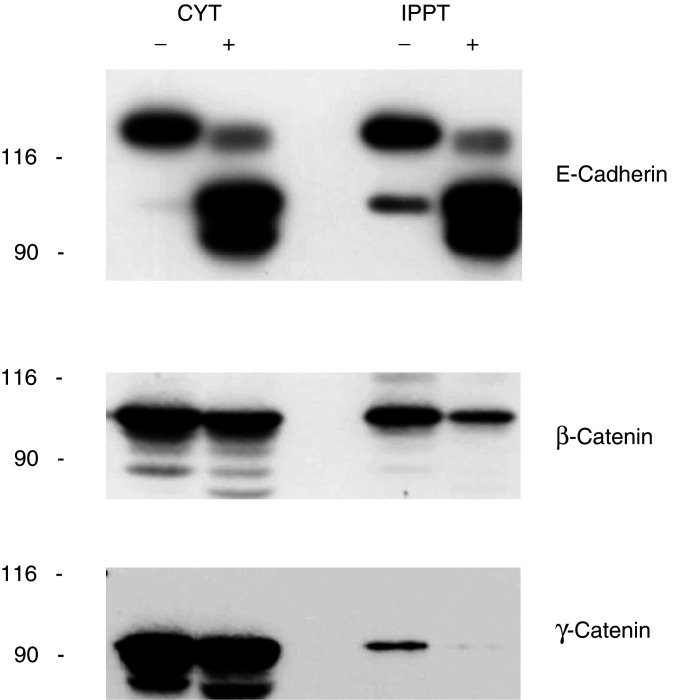
Effect of yessotoxin treatment of MCF-7 cells on the levels of *β*- and *γ*-catenins associated with E-cadherin. MCF-7 cells were treated with (+) or without (−) 1 nM YTX for 5 days at 37°C. At the end of the incubation, cells were processed to prepare cytosoluble extracts, which were subjected to immunoprecipitation using the HECD-1 anti-E-cadherin antibody, as described under Materials and Methods. Identical aliquots of cytosoluble extracts (CYT) and of immunoprecipitated material (IPPT) were subjected to SDS–PAGE and immunoblotting, using antibodies recognising the indicated proteins. The electrophoretic mobilities of *β*-galactosidase (116 kDa) and fructose-6-phosphate kinase (90 kDa) subunits, used as marker proteins running in a parallel lane, are indicated on the left.

The results we obtained are reported in Figure 7

, and show that the levels of *β*- and *γ*-catenins associated with E-cadherin in our immunoprecipitates were reduced by more than 60% in samples from YTX-treated cells, as compared to controls. Thus, cell treatment with YTX resulted in the disruption of the E-cadherin–catenin system in MCF-7 cells.

**Figure 8:**
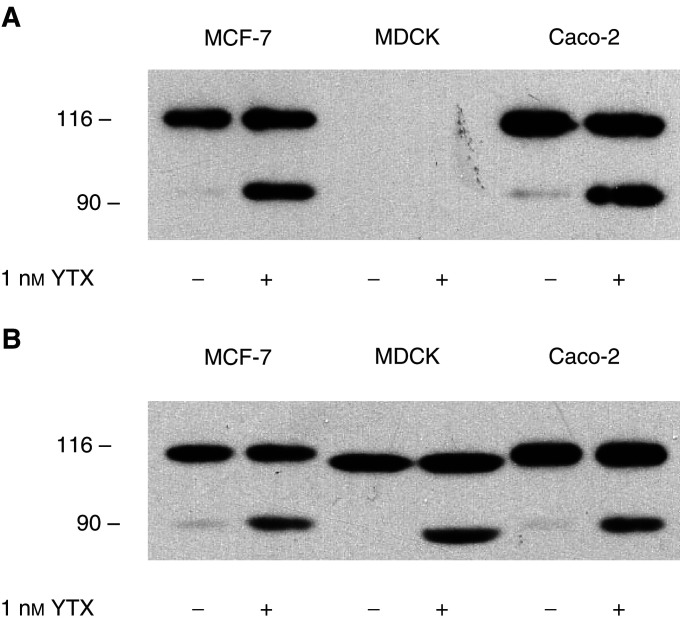
Effect of yessotoxin treatment of different epithelial cells on E-cadherin. MCF-7, Caco-2 and MDCK cells were incubated with 1 nM YTX for 24 h at 37°C. At the end of the incubation, cells were processed to prepare cytosoluble extracts, which were subjected to SDS–PAGE and immunoblotting, using the HECD-1 (**a**) and H-108 (**b**) anti-E-cadherin antibodies. The electrophoretic mobilities of *β*-galactosidase (116 kDa) and fructose-6-phosphate kinase (90 kDa) subunits, used as marker proteins running in a parallel lane, are indicated on the left.

This finding prompted us to probe whether YTX causes fragmentation of E-cadherin in other epithelial cells. We then extended our analyses to Caco-2 and MDCK cells, which were treated for 24 h with 1 nM YTX and cytosoluble extracts were analysed. Immunoblotting using the HECD-1 antibody led to the detection of ECRA_100_ in extracts from both MCF-7 and Caco-2 cells treated with YTX, but neither intact E-cadherin nor its ECRA_100_ fragment were detected in the extracts prepared from MDCK cells (Figure 8A

). The possibility that this result was due to species-specific differences in the amino-acid sequence of E-cadherin was then probed, and analyses were repeated using the H-108 anti-E-cadherin antibody. The results we obtained showed that YTX could induce accumulation of ECRA_100_ in the three epithelial cell lines (Figure 8B).

This finding led us to evaluate whether YTX could induce fragmentation of other members of the cadherin family. We then turned our attention to N-cadherin, which represents another well characterised component of classical cadherins, and is mainly expressed in cells of neural origin (Takeichi, 1990; Yap *et al*, 1997; Tepass *et al*, 2000).

**Figure 9:**
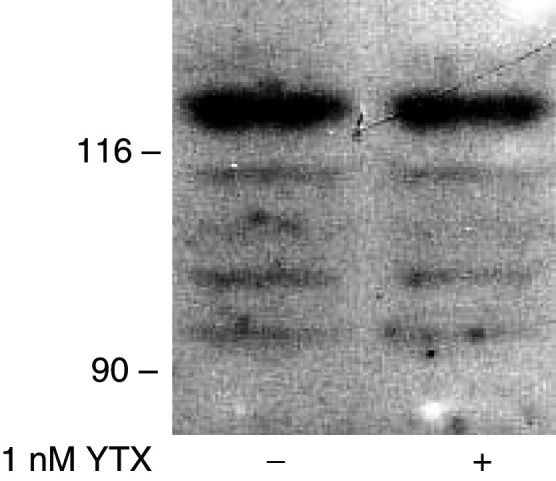
Effect of yessotoxin treatment of PC 12 cells on N-cadherin. Cells were treated with 50 nM NGF for 3 days before they were incubated with 1 nM YTX for 24 h at 37°C. At the end of the incubation, cells were processed to prepare cytosoluble extracts, which were subjected to SDS–PAGE and immunoblotting, using the anti-N-cadherin antibody. The electrophoretic mobilities of *β*-galactosidase (116 kDa) and fructose-6-phosphate kinase (90 kDa) subunits, used as marker proteins running in a parallel lane, are indicated on the left.

PC-12 cells, which express N-cadherin (Doherty *et al*, 1991) and represent an experimental system amenable of controlled proliferation and differentiation (Greene and Tischler, 1976; Marshall, 1995), were then used in our experiments. Cytosoluble extracts were prepared after PC-12 cells had been treated for 24 h with 1 nM YTX, and were subjected to immunoblotting using an anti-N-cadherin antibody. The results we obtained showed that the electrophoretic patterns of samples from YTX-treated cells were similar to those of extracts from control cells (Figure 9

).

In order to check whether those results might depend on the functional state of PC-12 cells, we repeated the experiment using culture conditions involving cells that had not received the differentiating stimulus of nerve growth factor, growing either in small clumps, or in monolayers (see Materials and Methods section). By immunoblotting using the anti-N-cadherin antibody, no differences in the electrophoretic patterns of N-cadherin were detected between control and YTX-treated samples in any of the experimental conditions we have employed (data not shown).

As YTX-induced fragmentation of E-cadherin was found to be independent of either the species or the tissues of origin of our cell lines (Figure 8), the results we obtained with N-cadherin could be due to differences among members of the superfamily of classic cadherins (Takeichi, 1990; Yap *et al*, 1997; Tepass *et al*, 2000).

**Figure 10:**
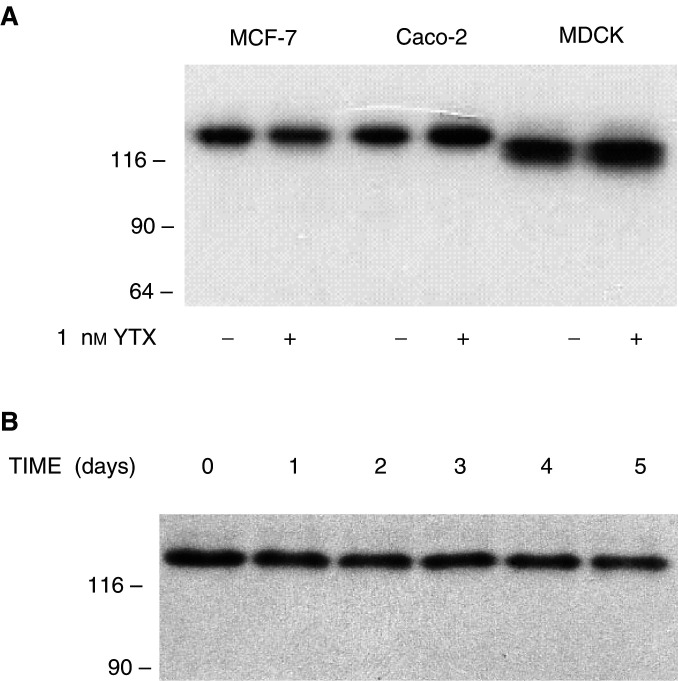
Effect of yessotoxin treatment of different epithelial cells on K-cadherin. (**A**) MCF-7, Caco-2 and MDCK cells were incubated with 1 nM YTX for 24 h at 37°C. At the end of the incubation, cells were processed to prepare cytosoluble extracts, which were subjected to SDS–PAGE and immunoblotting, using the anti-K-cadherin antibody. (**B**) Effect of prolonged yessotoxin treatment of MCF-7 cells on the cellular pool of K-cadherin. Cells were incubated with 1 nM YTX for the times indicated at 37°C, before being processed to prepare cytosoluble extracts, which were subjected to SDS–PAGE and immunoblotting, using the anti-K-cadherin antibody. The electrophoretic mobilities of *β*-galactosidase (116 kDa), fructose-6-phosphate kinase (90 kDa) and pyruvate kinase (64 kDa) subunits, used as marker proteins running in a parallel lane, are indicated on the left.

In order to probe the selectivity of YTX-induced response with regard to cadherin molecules, we analysed a third member of the cadherin family, K-cadherin (cadherin-6), in the MCF-7, Caco-2 and MDCK cells, which represents YTX-responsive systems. The results reported in Figure 6 were obtained when cytosoluble extracts were analysed by immunoblotting using an anti-K-cadherin, and showed that no differences were detectable in the electrophoretic patterns of K-cadherin in samples prepared from control and YTX-treated cells (Figure 10A

). This lack of sensitivity of the K-cadherin system to YTX was confirmed by the observation that 5-day treatment of MCF-7 cells with the toxin did not result in the detection of protein fragments, and led to limited (about 40%) losses of intact K-cadherin (Figure 10B).

## DISCUSSION

The analysis of the effects exerted by YTX on the structure of members of the cadherin superfamily of cell adhesion proteins has revealed that the toxin selectively induces the removal of the intracellular domain of E-cadherin, leading to the disruption of the E-cadherin–catenin system in MCF-7 cells. No gross alteration, instead, was detected in N- and K-cadherin, which are separate members of type I and type II classic cadherins, respectively (Tanihara *et al*, 1994; Nollet *et al*, 2000; Tepass *et al*, 2000).

The simplest explanation of these findings is that different members of the cadherin superfamily are not susceptible to the same kind of proteolytic attack. Alternatively, the possibility that the molecular machinery affected by YTX might not have the same features in different cellular systems should be considered. The first interpretation seems more likely, based on the finding that YTX causes fragmentation of E-cadherin, but not K-cadherin, in the same cell lines (Figures 8 and 10). Thus, the different sensitivity of E- and K-cadherin to YTX would further confirm the conclusion that the turnovers of the two proteins are independently regulated by both endogenous and exogenous stimuli (Stewart *et al*, 2000).

The accumulation of ECRA_100_ on the first day of YTX treatment of MCF-7 cells did not cause their massive detachment from culture dishes. The maintenance of adhesive properties in YTX-treated cells could be due to the fact that the initial accumulation of ECRA_100_ is not apparently accompanied by an extensive loss in the cellular content of intact E-cadherin (Figure 2), so that an increase in the total immunoreactivity of samples is observed in MCF-7 cells (Pierotti *et al*, 2003). This interpretation would be supported by the finding that no ECRA_100_ could be detected in the culture media of YTX-treated cells. Furthermore, YTX did not cause a massive redistribution of E-cadherin between Triton X-100 soluble and insoluble fractions (Figure 6), which have been proposed to include the molecules involved in weak and strong cell adhesions, respectively (Shore and Nelson, 1991; Chitaev and Troyanovsky, 1998; Takeda *et al*, 1999).

It should be noted, however, that K-cadherin was expressed in our experimental systems, and that our analyses showed that the general structure and cellular content of K-cadherin were not significantly affected by YTX treatment in our cell lines. Thus, we cannot exclude that an intact K-cadherin pool might compensate for an altered E-cadherin system in both normal (MDCK) and tumour (MCF-7 and Caco-2) cells. In any case, K-cadherin alone cannot substitute for E-cadherin, as cell detachment from culture dishes was observed after prolonged YTX treatment of MCF-7 cells (Figure 3), when the cellular pool of K-cadherin was not disrupted yet (Figure 10)

To the best of our knowledge, this is the first report describing the expression of K-cadherin in breast cancer cells. Indeed, we have been using MCF-7 cells as a model system to evaluate the alteration of molecular mechanisms involved in signal transduction (Rossini *et al*, 1999; Malaguti and Rossini, 2002). Thus, the finding that different epithelial cells can be included among the targets of an algal toxin contaminating products destined for human consumption calls for a deeper insight into the possibility that long-term effects might be caused in the intact organism by ingestion of low doses of YTX.

The maximal content of YTX contaminating seafood that can be placed on the market is presently regulated by the legislation of the European Communities, and is represented by 1 mg of YTX equivalents per kilogram of the edible parts of bivalve molluscs and other species (European Communities, 2002). This limit has been set taking into consideration the very low oral toxicity of YTX and its analogues, based on the analyses of acute responses in mice (Aune *et al*, 2002).

Our observations on the disruption of the E-cadherin system caused by low concentrations of YTX (less than 1 *μ*g l^−1^) in cultured cells should justify other studies aimed at evaluating whether this compound might induce some delayed type of adverse effect(s) in intact organisms. This would be particularly relevant with regard to the intestine epithelium, as the E-cadherin pool of Caco-2 cells, which has been derived from a human colorectal adenocarcinoma (Fogh *et al*, 1977), is altered by YTX.

The possible long-term effects of YTX in intact organism, based on the results we obtained in the present study, could include the disruption of the tumour-suppressing function of E-cadherin. More precisely, our data show that YTX causes both a decrease in cell adhesion (Figure 3) and in the levels of *β*- and *γ*-catenins associated with E-cadherin (Figure 7).

It is recognised that a loss of cell adhesion, as a consequence of altered E-cadherin participates in tumour spreading and metastasis formation (Birchmeier and Behrens, 1994; Mareel *et al*, 1997; Christofori and Semb, 1999; Beavon, 2000). Under our experimental conditions, the YTX-induced detachment of cells from culture dishes (Figure 3) was accompanied by cell growth arrest (Figure 4). The proliferation of MCF-7 cells, however, has been shown to be halted when the cellular E-cadherin pool is reduced, and the recovery of normal cell growth is observed after the repletion of normal levels of E-cadherin (Malaguti and Rossini, 2002). Thus, our data could support the working hypothesis that YTX might facilitate metastasis formation.

In any case, this interpretation should not exclude that YTX might have other effects by interfering with the Wnt signalling machinery (Peifer and Polakis, 2000; Zhurinsky *et al*, 2000; Taipale and Beachy, 2001), as the loss in *β*- and *γ*-catenin interacting with E-cadherin has been found to occur without a concomitant decrease in the total cytosoluble pool of *β*- and *γ*-catenin (Figure 7). Thus, cell treatment with YTX would result in an increased pool of free *β*- and *γ*-catenin, which could then interfere with the Wnt signalling pathway.

In conclusion, the results of the present study show that YTX induces an alteration of E-cadherin, which determines the disruption of the E-cadherin–catenin system and has the potential to disrupt the tumour-suppressive role of E-cadherin.
